# OVERVIEW OF NIA BUDGET AND RESEARCH

**DOI:** 10.1093/geroni/igae098.0330

**Published:** 2024-12-31

**Authors:** Richard Hodes

**Affiliations:** National Institute on Aging, Baltimore, Maryland, United States

## Abstract

Dr. Hodes will discuss the NIA budget, overall research priorities of the Institute, NIA-funded research advances, and key programs and initiatives.

